# Preoperative Metastatic Brain Tumor-Associated Intracerebral Hemorrhage Is Associated With Dismal Prognosis

**DOI:** 10.3389/fonc.2021.699860

**Published:** 2021-09-14

**Authors:** Motaz Hamed, Niklas Schäfer, Christian Bode, Valeri Borger, Anna-Laura Potthoff, Lars Eichhorn, Frank A. Giordano, Erdem Güresir, Muriel Heimann, Yon-Dschun Ko, Jennifer Landsberg, Felix Lehmann, Alexander Radbruch, Elisa Scharnböck, Christina Schaub, Katjana S. Schwab, Johannes Weller, Ulrich Herrlinger, Hartmut Vatter, Patrick Schuss, Matthias Schneider

**Affiliations:** ^1^Department of Neurosurgery, Center of Integrated Oncology (CIO) Bonn, University Hospital Bonn, Bonn, Germany; ^2^Division of Clinical Neuro-Oncology, Department of Neurology, Center of Integrated Oncology (CIO) Bonn, University Hospital Bonn, Bonn, Germany; ^3^Department of Anesthesiology and Intensive Care, University Hospital Bonn, Bonn, Germany; ^4^Department of Radiation Oncology, Center of Integrated Oncology (CIO) Bonn, University Hospital Bonn, Bonn, Germany; ^5^Department of Oncology and Hematology, Center of Integrated Oncology (CIO) Bonn, Johanniter Hospital Bonn, Bonn, Germany; ^6^Department of Dermatology and Allergy, Center of Integrated Oncology (CIO) Bonn, University Hospital Bonn, Bonn, Germany; ^7^Department of Neuroradiology, Center of Integrated Oncology (CIO) Bonn, University Hospital Bonn, Bonn, Germany; ^8^Department of Internal Medicine III, Center of Integrated Oncology (CIO) Bonn, University Hospital Bonn, Bonn, Germany

**Keywords:** hemorrhage, brain metastases, overall survival, hemorrhagic transformation, cranial surgery

## Abstract

**Object:**

Intra-tumoral hemorrhage is considered an imaging characteristic of advanced cancer disease. However, data on the influence of intra-tumoral hemorrhage in patients with brain metastases (BM) remains scarce. We aimed at investigating patients with BM who underwent neurosurgical resection of the metastatic lesion for a potential impact of preoperative hemorrhagic transformation on overall survival (OS).

**Methods:**

Between 2013 and 2018, 357 patients with BM were surgically treated at the authors’ neuro-oncological center. Preoperative magnetic resonance imaging (MRI) examinations were assessed for the occurrence of malignant hemorrhagic transformation.

**Results:**

122 of 375 patients (34%) with BM revealed preoperative intra-tumoral hemorrhage. Patients with hemorrhagic transformed BM exhibited a median OS of 5 months compared to 12 months for patients without intra-tumoral hemorrhage. Multivariate analysis revealed preoperative hemorrhagic transformation as an independent and significant predictor for worsened OS.

**Conclusions:**

The present study identifies preoperative intra-tumoral hemorrhage as an indicator variable for poor prognosis in patients with BM undergoing neurosurgical treatment.

## Introduction

Hemorrhage in brain metastases (BM) is not rare, especially in the case of BM in melanoma patients (1, 2). The incidence of intracerebral/intra-tumoral hemorrhage caused by cerebral neoplasms may exceed 20% (2–5). With technical advances in imaging, microbleeds within tumors are becoming increasingly visualized even when they may have remained clinically inapparent (6, 7). On the other hand, acute hemorrhage may also trigger new neurologic symptoms or their aggravation in a considerable number of patients (2). This sudden event may lead to expedited access to diagnostic testing for BM, which may be associated with a more favorable disease course due to potentially earlier initiation. However, increased aggressiveness and pathological remodeling processes within the tumor tissue are also debated as potential mechanisms for hemorrhage (8). This notion is corroborated by reports on low tumor control rates after stereotactic radiotherapy (9, 10). The circumstance of inconsistent/different post-treatment modality may be a substantial contributor to a poorer outcome being reported in some cases (11). Nonetheless, important information on the influence of the observed hemorrhage on the prognosis of patients with BM remains scarce. In the present study, we reviewed and analyzed all patients who underwent surgical treatment for BM from extracranial primaries at our institution. By investigating the impact of this factor on survival before undergoing surgical resection, we hope to circumvent a possible surrogate effect that could result from radiation therapy alone to a hemorrhagic transformed lesion. Therefore, the primary study objective was to determine the influence of hemorrhagic transformation as an preoperatively easily collectable MRI-morphological parameter on overall survival for BM requiring surgery.

## Methods

### Patients

All patients with surgical management and subsequent histopathological evidence of BM in the authors’ institution were entered into a computerized database (SPSS, version 25, IBM Corp., Armonk, NY). Beforehand, this study had been approved by the institutional ethics committee. Institutional ethics committee approval was obtained for this study. Individual treatment decisions were determined at the first presentation of the patient and during subsequent follow-up through weekly institutional interdisciplinary tumor advisory board meetings for the central nervous system, as previously described (4).

Information, including patient characteristics, radiological features, preoperative laboratory values and functional neurological status at admission was collected and further analyzed. Hemorrhagic metastases were those that were either clearly identified on imaging by blood fluid levels, in cases where MRI sequencing on T1 pre-contrast sequences suggested the presence of blood products, or lesions suspicious for bleeding were identified on susceptibility weighted imaging (SWI). Tumor volumes were volumetrically assessed manually in T1 weighted MRI sequences using commercially available software (TumorTracking Tool, IntelliSpace Portal 5.0, Philips, Best, the Netherlands). The Karnofsky performance score (KPS) was used to evaluate patients according to their neurological functional status preoperatively. For further analysis, the results were dichotomized and thus a KPS ≥70 was defined as favorable outcome. Regarding the preoperative classification according to the American Society of Anesthesiologists (ASA), the included patients were divided into two groups: preoperative ASA 1 or 2 versus preoperative ASA ≥ 3. To estimate the preoperative comorbidity burden of surgically treated patients with BM, the Charlson Comorbidity Index (CCI) was assessed using the preoperative information. Here, patients were divided into two groups according to their burden: CCI ≤ 10 versus CCI > 10, as previously defined (4).

Overall survival (OS) was measured from the day of BM surgery until death or last observation. Patients in whom no further follow-up information was available due to further off-site treatment were excluded from further analysis. All parameters were compared in terms of OS.

### Statistics

Data analysis was performed using the computer software package SPSS (version 25, IBM Corp., Armonk, NY). Unpaired categorical and binary variables were analyzed in contingency tables using the Fisher’s exact test. The Mann-Whitney U-test was chosen to compare continuous variables as the data were mostly not normally distributed. OS was analyzed by the Kaplan-Meier method. With reference to Heinze and Dunkler (12) preoperatively collectable parameters with known prognostic influence based on the literature as well as hemorrhagic transformation were entered in a multivariate analysis using the Cox proportional hazard model. Results with p<0.05 were considered statistically significant.

## Results

### Patient Characteristics

Between 2013 and 2018, a total of 382 patients with BM were surgically treated at the authors’ neuro-oncological center. 25 patients were excluded from further analysis after careful review of clinical records due to lack of further follow-up information. Therefore, 357 patients with surgically treated BM were included in further analysis. The median age was 65 years (range 20-91 years). At admission, patients presented with a median KPS score of 80. Median overall survival for patients with surgically treated BM was 10 months (95% CI 8.1-11.9).

26 out of 357 patients with surgically-resected BM (7%) were treated with adjuvant immuno- and/or chemotherapy only, 66 patients (18%) with adjuvant radiotherapy only and 233 patients (62%) with adjuvant combined chemo-, immuno- and radiotherapy. Patients with postoperative adjuvant oncological therapy exhibited a mOS of 12 months compared to 1 month for patients without postoperative adjuvant treatment (p<0.0001). 20 out of 244 patients with solitary BM (8%) received adjuvant immuno- and/or chemotherapy, 50 patients (20%) received adjuvant radiotherapy (among them 9 patients with whole brain radiation) and 146 patients (60%) were treated with a combination of adjuvant radio-, immuno- and chemotherapy. 28 patients (11%) received no adjuvant oncological therapy. 70 out of 133 patients (53%) with multiple BM received postoperative whole brain RT. See **Table 1** for more patient-specific details.

**Table 1:** Patient characteristics.

	Patients with surgically treated BM (n = 357)
median age at surgery (yrs)	65
female sex	175 (49%)
preoperative KPS ≥ 70	309 (87%)
Primary site of cancer	
Lung	154 (43%)
Breast	45 (13%)
Melanoma	38 (11%)
Other	120 (34%)
CCI > 10	185 (52%)
ASA ≥ 3	200 (56%)
Preoperative anticoagulant medication	72 (20%)
multiple BM	113 (32%)
Hemorrhagic BM	122 (34%)
median OS (mo)	10 (95% CI 8.1-11.9)

BM, brain metastases; yrs, years; KPS, Karnofsky Perfomance Scale; CCI, Charlson Comorbidity Index; ASA, American Society of Anesthesiologists; mo, months.

### Influence of Intra-Tumoral Hemorrhagic Transformation/Hemorrhage

Overall, 122 patients (34%) suffered from preoperative hemorrhagic transformation of the surgically treated BM (**Table 2**). Patients with hemorrhagic BM were significantly older compared to patients without BM-associated hemorrhage at the time of surgery (p=0.003). Patients with intratumoral hemorrhage exhibited a median tumor volume of 24 ml (95% CI 18-30) compared to 14 ml (95% CI 10-17) for patients without preoperative intratumoral hemorrhage (p=0.002). Spearman correlation analysis could not find a strong correlation between tumor volume and OS (Spearman’s r = -0.12). Patients with hemorrhagic BM presented significantly more often with preoperative KPS < 70 compared to patients with non-hemorrhagic BM (p=0.02). Regarding the site of origin of the cancer, hemorrhagic transformed BM have been observed preoperatively significantly more often in patients with lung cancer (p=0.01) and patients with melanoma (p<0.0001). In contrast, patients with metastatic breast cancer presented less often with hemorrhagic transformed BM (p=0.04). Patients with higher preoperative comorbidity burden (CCI > 10) were more likely to suffer from preoperative bleeding into the BM to be resected than patients with lower burden of comorbid diseases (p=0.001). Patients with hemorrhagic BM received significantly more frequent preoperative administration of anticoagulant medication compared to patients without hemorrhagic BM (p=0.005). 26 out of 122 patients (21%) with preoperative intratumoral haemorrhage suffered from local recurrence of the metastatic lesion in the later course of cancer disease compared to 71 out of 253 patients (28%) without preoperative intratumoral haemorrhage (p=0.2).

**Table 2:** Characteristics of hemorrhagic brain metastases.

	Patients w/o hemorrhagic BM (n = 235)	Patients with hemorrhagic BM (n = 122)	p-value
median age at surgery (yrs, IQR)	62 (55-71)	68 (59-75)	0.003
female sex	114 (49%)	68 (56%)	n.s.
preoperative KPS ≥ 70	211 (90%)	98 (80%)	0.02
Primary site of cancer			
Lung	113 (48%)	41 (34%)	0.01
Breast	36 (15%)	9 (7%)	0.04
Melanoma	10 (4%)	28 (23%)	<0.0001
Other	76 (32%)	44 (36%)	n.s.
CCI > 10	107 (46%)	78 (64%)	0.001
ASA ≥ 3	123 (52%)	77 (63%)	n.s.
Preoperative anticoagulant medication	37 (16%)	35 (29%)	0.005
multiple BM	72 (31%)	41 (34%)	n.s.
median OS (mo)	12 (95% CI 9.5-14.5)	5 (95% CI 3.6-6.4)	0.002

BM, brain metastases; yrs, years; IQR, interquartile range; KPS, Karnofsky Perfomance Scale; CCI, Charlson Comorbidity Index; ASA, American Society of Anesthesiologists; OS, overall survival; mo, months; CI, confidence interval; n.s., not significant.

**Figure 1:**
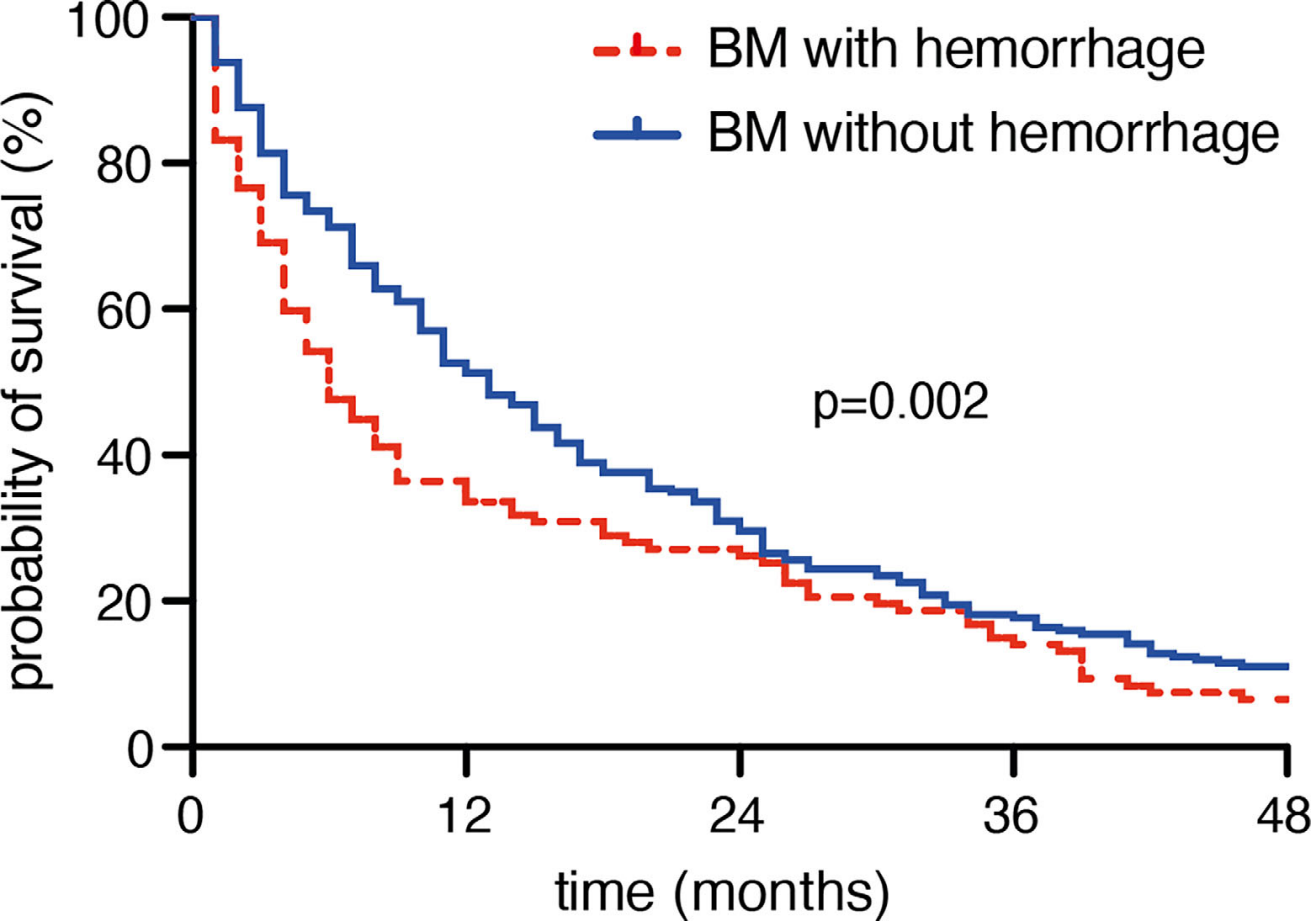
Preoperative hemorrhage is associated with poor survival in patients with brain metastases.

Patients with a preoperative hemorrhagic transformed BM achieved a median OS of 5 months (95% CI 3.6-6.4), whereas patients with BM but without an apparent intra-tumoral hemorrhage achieved a median OS of 12 months (95% CI 9.5-14.5; p=0.002; **Figure 1**).

### Multivariate Analysis

A Cox regression analysis was performed in order to identity factors influencing OS in patients with surgically treated BM. The multivariate analysis revealed “age > 65 years” (p=0.005, HR 1.4, 95% CI 1.1-1.8), “preoperative KPS < 70” (p=0.005, HR 1.6, 95% CI 1.2-2.3), “multiple BM” (p<0.0001, HR 1.7, 95% CI 1.3-2.2), as well as “preoperative hemorrhagic transformation of BM” (p=0.009, HR 1.4, 95% CI 1.1-1.8) as independent and significant predictors for dismal overall survival.

## Discussion

Intra-tumoral hemorrhage in patients with metastatic intracranial lesions is a recognized characteristic that seems to occur in about 24% of all BM (2, 13). This number was found to be reproducible in the present study. Furthermore, in this selected patient collective of patients with surgically treated BM, preoperative intra-tumoral hemorrhage is shown to be a significant predictor of poor prognosis.

Various theories for the origin of intra-tumoral hemorrhage have been discussed. These include tumor-induced endothelial proliferation with vascular obliteration, vessel compression and/or distortion due to rapid aggressive tumor growth, as well as tumorous vessel wall invasion, and also elevated venous pressure in response to increased local intracranial pressure (2, 8). These histopathological interpretations seem to indicate an increased aggressiveness within the tumor lesion in patients with BM and intra-tumoral hemorrhage. This aspect seems to be reflected in the fact that patients with intra-tumoral hemorrhage had a worse prognosis in the present study.

Even in the case that surgical resection is not chosen as the method of first choice, intra-tumoral hemorrhage in BM seems to be associated with worse survival/outcome in other studies as well. Thus, a recent study by Bauer-Nilsen and colleagues has demonstrated that in cases of hemorrhagic BM, local control by radiotherapy is less likely to be achieved compared with BM without hemorrhagic transformation (14).

Nevertheless, the mere survival of patients with and without intra-tumoral hemorrhage has to be illuminated in more detail on the basis of the different characteristics. Patients with hemorrhagic transformation of BM are significantly older at the time of surgery compared to patients without such hemorrhage. Age itself seems to play an equally important role with regard to OS in patients with surgically treated BM, as shown by the present multivariate analysis. Apart from intra-tumoral hemorrhage, (older) age is an important prognostic factor for overall survival in patients with BM (15–17). Older age might also lead to an increased correlating burden of comorbidities. Again, a higher preoperative CCI value is evident in the present patient collective with intra-tumoral hemorrhage. An increased burden of comorbid concomitant diseases is also associated with a higher incidence of postoperative complications, which can significantly influence the further survival of the affected patient (4, 18). According to the definition of the CCI used here, existing diseases that require anticoagulant medication are also included (e.g., myocardial infarction, history of transient ischemic attacks) (19). Thus, it should come as no surprise that patients on anticoagulant medication are significantly more likely to present for surgical resection with intra-tumoral hemorrhage in the present series. On the other hand, cancer patients are more at risk for thromboembolic events. However, the rate of additional intra-tumoral hemorrhage does not seem to be influenced by the administration of low-molecular-weight heparin in patients with BM (20). Furthermore, indicated therapeutic anticoagulation in patients with BM does not appear to confer an increased risk of intracerebral hemorrhage/intra-tumoral hemorrhage based on the existing evidence (21). Moreover, the presence and severity of preoperative intratumoral hemorrhage might also depend on the entity of the underlying cancer disease. In line with the trend in the present series, melanoma has been reported to constitute a tumor entity with hemorrhagic transformation in the earlier course of disease (22). Further tumor entities like renal cell carcinoma are known to exhibit elevated levels of intratumoral hemorrhage (23) and might therefore not only lead to a correlation between tumor entity and survival, but also between the level of preoperative hemorrhage and survival. Further studies will be needed in order to more sufficiently address these potential prognostic MRI morphologic characteristics.

Nevertheless, in contrast to this possible causal linkage, the present multivariate cox analysis reveals a clear and independent influence of intra-tumoral hemorrhage on OS in patients with BM requiring surgical treatment. However, this fact should not discourage, but on the contrary: it should sensitize the perception regarding this seemingly elementary influence of intra-tumoral hemorrhage enabling a timely planning of the further therapy as well as a more precise information of patients/relatives about the eventual course of the disease as early as in the preoperative phase. In particular, due to a lower efficacy of radiosurgery of hemorrhagic BMs, potential improvement of neurological deterioration as well as reduction of tumor burden, surgical resection should remain as a treatment option if feasible (13, 14). However, because of the potential accompanying specific factors, an individual and in particular interdisciplinary evaluation of affected patients with BM indispensably remains an essential part of patient-centered management.

## Limitations

Besides the inherent limitation of the retrospective design of this study, another relevant shortcoming is the assessment of hemorrhagic characteristics. These features were not centrally reviewed, leading to a potential interpreter bias. Despite a moderate sample size, further subgroup analyses are limited because of the then smaller number of hemorrhagic events.

## Conclusions

In the present study, preoperative evidence of intra-tumoral hemorrhage was identified as indicators of poor prognosis in patients with BM undergoing neurosurgical treatment. In addition to improved counseling of affected patients, this finding also enables timely and individualized planning of further therapy in the context of interdisciplinary decision making in patients with BM.

## Data Availability Statement

The original contributions presented in the study are included in the article/supplementary material. Further inquiries can be directed to the corresponding author.

## Ethics Statement

The studies involving human participants were reviewed and approved by Ethics Committee University Hospital Bonn. Written informed consent for participation was not required for this study in accordance with the national legislation and the institutional requirements.

## Author Contributions

Conceptualization: MoH, PS and MS. Methodology: MuH, PS and MS. Validation: MoH, NS, UH, PS and MS. Formal analysis: MoH, NS, PS and MS. Writing – original draft preparation: MoH, PS and MS. Writing – review and editing: MoH, NS, CB, VB, LE, FG, EG, MoH, Y-DK, JL, FL, AR, ES, CS, KS, JW, UH, HV, PS and MS. Visualization: PS and MS. Supervision: UH, HV, PS and MS. Project administration: PS and MS. All authors contributed to the article and approved the submitted version.

## Conflict of Interest

The authors declare that the research was conducted in the absence of any commercial or financial relationships that could be construed as a potential conflict of interest.

## Publisher’s Note

All claims expressed in this article are solely those of the authors and do not necessarily represent those of their affiliated organizations, or those of the publisher, the editors and the reviewers. Any product that may be evaluated in this article, or claim that may be made by its manufacturer, is not guaranteed or endorsed by the publisher.
